# Estimation accuracy in the psychological sciences

**DOI:** 10.1371/journal.pone.0207239

**Published:** 2018-11-26

**Authors:** Clintin P. Davis-Stober, Jason Dana, Jeffrey N. Rouder

**Affiliations:** 1 Department of Psychological Sciences, University of Missouri, Columbia, MO, United States of America; 2 Yale School of Management, Yale University, New Haven, CT, United States of America; 3 Department of Cognitive Sciences, University of California, Irvine, Irvine, CA, United States of America; Roswell Park Cancer Institute, UNITED STATES

## Abstract

Sample means comparisons are a fundamental and ubiquitous approach to interpreting experimental psychological data. Yet, we argue that the sample and effect sizes in published psychological research are frequently so small that sample means are insufficiently accurate to determine whether treatment effects have occurred. Generally, an estimator should be more accurate than any benchmark that systematically ignores information about the relations among experimental conditions. We consider two such benchmark estimators: one that randomizes the relations among conditions and another that always assumes no treatment effects. We show conditions under which these benchmark estimators estimate the true parameters more accurately than sample means. This perverse situation can occur even when effects are statistically significant at traditional levels. Our argument motivates the need for regularized estimates, such as those used in lasso, ridge, and hierarchical Bayes techniques.

## Introduction

We investigate the problem of estimation accuracy for fundamental research designs in psychology and related areas. Specifically, researchers often measure some dependent variable from subjects who are assigned to different experimental conditions. Typically, researchers take the mean of the dependent variable in each condition, and try to determine whether any differences in the means are due to treatment or noise.

We approach this old problem in a new way by comparing the accuracy of sample means—that is, how closely they track the population means—to two benchmark estimators. These benchmarks systematically ignore important features of the data, namely which means are larger than others and by how much. As such, these benchmarks are absurd, at least for the purpose of estimating treatment effects. We argue simply that a researcher should be alarmed if these absurd benchmarks were actually closer, on average, to the “truth,” i.e., the set of corresponding population means, than the corresponding sample means. Yet, as we demonstrate, research in many areas of psychology typically employs sample and effect size combinations such that these absurd benchmarks are indeed closer to the truth than sample means. Notably, achieving statistical significance does not preclude being in this situation. Our arguments achieve three goals:

- we provide new and more principled arguments for determining sufficient sample sizes in behavioral science experiments,
- we call for the widespread adoption of modern estimation methods for estimating population means, e.g., Bayesian methodologies.
- we provide a more nuanced perspective on the replication crisis in the behavioral sciences [1, 2], one based on the limits of estimation rather than binary decisions based on null hypothesis testing.

Our two benchmark estimators are not intended to actually be used by behavioral scientists to estimate relationships among population means. Rather, they are a tool for setting up a minimum standard of estimation accuracy that a reasonable experimenter would want to surpass. One of our benchmark estimators randomizes the relations among experimental conditions, both in terms of which means might be larger than others and also the relative size of the differences between them. The other benchmark simply assumes that there are no condition effects, no matter the data. Whether these absurd benchmarks are more precise than sample means depends on the true size of the treatment effect and the sample size.

We demonstrate that these absurd estimators outperform sample means when the true effects and sample sizes are modest in size. We provide exact expressions of how small these values have to be, and find that commonly used sample sizes in many areas of psychology are insufficient to insure that sample means outperform these absurd estimators. Based on the assumption that researchers want their sample means to be more accurate than absurd approaches to science that obscure or scramble treatment effects, we provide new standards for minimum sample sizes researchers should obtain before interpreting their results. Generally, this approach would call for larger sample sizes. Researchers can avoid these problems altogether by replacing the use of sample means with modern estimators, including Bayesian methods, lasso and ridge techniques. Insofar as our benchmarks are compelling, our guidelines do not reflect hypotheses, models, or arbitrary decision criteria.

Our paper is not the first to raise the question of poor estimation accuracy within the field of psychology. Many authors have found that studies may typically yield inaccurate parameter estimates that are highly variable across replicates, leading to contradictory findings within the literature [3] as well as core findings that do not replicate [1, 4]. We further this discussion by taking steps toward precisely defining minimally acceptable estimation accuracy. One could conclude that a treatment effect exists under some decision criterion, for example null hypothesis testing, using estimates that are unacceptably accurate. Our paper extends current approaches to quantifying unacceptable estimation accuracy within the social sciences [2] by comparing a status quo method to a concrete set of benchmarks.

### The use of accuracy benchmarks

It is a common scientific practice to adopt accuracy benchmarks. For example, experimental chemists and physicists specify precisely how accurate equipment must be to conduct meaningful experiments. Computer scientists use performance benchmarks when training classifiers to categorize objects, some as simple as a random classifier that guesses to which category an object belongs. A natural way to think about adequate estimation accuracy is to consider minimum benchmark values that should be exceeded. Though it may be unfamiliar to describe them in these terms, sample means are a piece of equipment as well, and it is natural to ask whether they are tuned accurately enough for answering one’s scientific question.

At first blush, it may seem that mainstream psychological research has already addressed the problem of standards for estimation accuracy. Null hypothesis statistical testing, despite its myriad criticisms [5–7], provides an objective rule for concluding whether a result was an accident of sampling error. Confidence intervals, recommended by the APA when reporting significance values [8], characterize the amount of uncertainty in estimates. These techniques have meaningful interpretations, but we seek a benchmark that is free of hypotheses, model assumptions and/or conventional (but arbitrary) settings. For example, *p*-value thresholds of.05 or.01 to reject null hypotheses, while precise, are arbitrary and not derived from any normative argument about what the likelihood of data under the null hypothesis *should* be given the question of interest. Rosnow and Rosenthal [9] famously emphasized the lack of an ontological basis for fixed alpha testing by noting “…surely, God loves the.06 nearly as much as the.05.” Similarly, what one person may find to be a narrow enough confidence interval may be unacceptably wide to someone else. Our goal is to derive precise and non-arbitrary standards for estimation accuracy.

One type of meaningful performance benchmark for establishing whether we have learned something is an *uninformed* benchmark like random guessing. For example, if one has truly learned something about a subject matter, one should score better, on average, than random guessing on a valid multiple choice exam. As another example, consider evaluating the performance of a mutual fund manager. If the fund returned 8% on its investments last year, we may wonder whether 8% was merely lucky or significantly different from zero. It is perhaps more informative to ask whether the fund outperformed an unweighted index of the entire market, because the index represents the expected performance of random stock picking. If a mutual fund performs no better than a stockpicker who throws darts at the names of listings, then we cannot yet conclude that the fund’s manager has any skill in picking stocks.

In the context of running experiments and determining whether the treatments had an effect, an uninformed benchmark against which to compare sample means would be one that ignores information about treatment effects. Such a benchmark could consider absolute levels when estimating population means, like the observed grand mean across conditions, but would not attend to information about how the conditions compared with each other. Using this approach to set standards of estimation accuracy has advantages over paradigms like null hypothesis testing. Importantly, this approach assume nothing about the generating distribution of the data. Thus, all conclusions are distribution-free and conclusions about estimation accuracy are not conflated with the goodness-of-fit of one’s statistical model.

### Motivating example

Assume that we have *p*-many population means, *μ*_*i*_, *i* ∈ {1, 2…, *p*}, and that that ${\hat{\mu }}_{i}$ is an estimate of the corresponding population mean *μ*_*i*_. Let $\hat{\mu }={\hat{\mu }}_{i},i\in \left\{1,2\ldots ,p\right\}.$ Throughout, we use mean squared error (MSE) as a metric of estimation accuracy. The MSE of an estimator $\hat{\mu }$ is defined as

$$\begin{array}{c} MSE_{\hat{\mu }}≔E\left[\sum_{i=1}^{p}{\left({\hat{\mu }}_{i}-\mu_{i}\right)}^{2}\right], \end{array}$$

where *E*[⋅] is the usual expectation operator of a random variable. MSE has some nice properties. It is well known that, for any estimator,

$$\begin{array}{c} E\left[\sum_{i=1}^{p}{\left({\hat{\mu }}_{i}-\mu_{i}\right)}^{2}\right]=\sum_{i=1}^{p}Var\left({\hat{\mu }}_{i}\right)+\sum_{i=1}^{p}{\left(E\left[{\hat{\mu }}_{i}\right]-\mu_{i}\right)}^{2}, \end{array}$$

where $Var({\hat{\mu }}_{i})$ denotes the variance of ${\hat{\mu }}_{i}$. Said simply, the MSE of any estimator can be described as a sum of the estimator’s total variance and its squared bias, $\sum_{i=1}^{p}{\left(E\left[{\hat{\mu }}_{i}\right]-\mu_{i}\right)}^{2}$. The key insight to be gained from this decomposition is that it is possible for a biased estimator to be more accurate (smaller MSE) than an unbiased one if the biased estimator has less variance.

**Fig 1:**
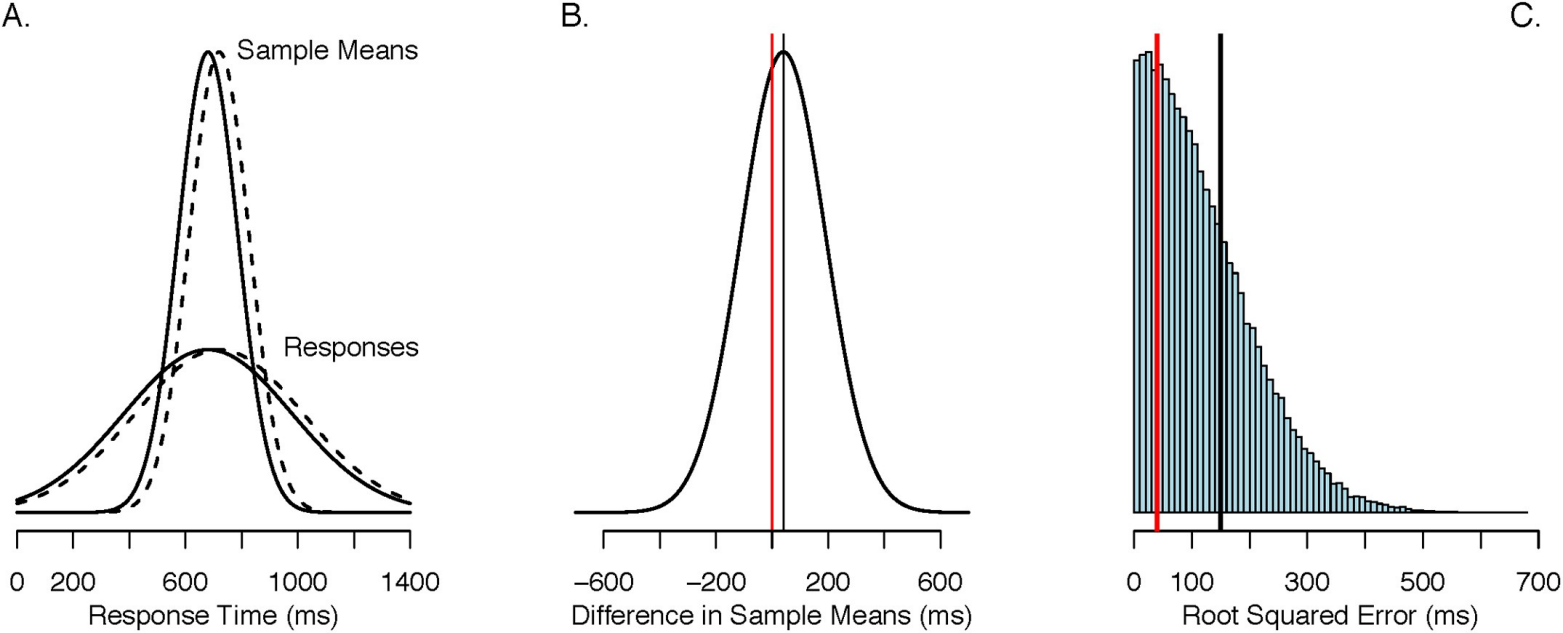
Estimators and error. **A**. Distributions of observations and sample means across 8 observations for the African American actor condition (solid line) and White actor condition (dashed-line). **B**. Distribution of the measurement tool, the difference in sample means. The vertical line is the zero estimator. **C**. Distribution of the absolute error for the sample mean measurement tool. The root-mean-squared error is 7 times greater for the sample mean tool than the zero estimator.

To illustrate this idea and locate our arguments within a concrete example, we consider Correll, Park, Judd, and Wittenbrink’s [10] race-based weapons priming task. In this task, participants were shown pictures of actors holding guns or similarly-shaped objects such as cell phones. The task was to decide whether the held object was a gun. Correll et al. found that participants respond more often and more quickly that the object is a gun when it is held by an African American actor than a White actor. For the sake of this example, let’s assume the effect is both real and small, such that participants respond 40 ms faster, on average, when the gun is held by African American actors. Let each observation in the African American actor condition be normally distributed with *μ*_*A*_ = 680 ms and *σ* = 300 ms; likewise, let each observation in the White actor condition be normally distributed with *μ*_*W*_ = 720 ms and *σ* = 300 ms. The response-time distributions for the African American actor condition (solid line) and White actor condition (dashed line) are displayed in Fig 1A. The difference of 40 ms is quite small compared to the variability. Fortunately, researchers collect multiple observations from each condition. Let’s take for example the case where the researcher collects eight observations for each actor condition, for a total of sixteen observations. We calculate the sample mean for each condition, comprised of eight observations each, and the distribution of these sample means is plotted in Fig 1A as well. The reduction in variance is obvious.

We are interested in the difference between the race condition sample means, and this difference serves as an estimate of the true effect. Fig 1B shows the distribution of this measurement, and it is centered at 40 ms, the true effect (black vertical line), but the resolution is quite poor, i.e., the width of this distribution is large. We can ask whether the difference between the sample means has sufficient “resolution” to estimate the true effect in this environment.

One way of assessing the accuracy of the sample mean difference is to compare it to an alternative method. The method we choose is crude and not useful in general. It returns the grand mean across all 16 observations as the measurement for each race condition, and the measurement of the true effect is identically zero. This estimator is certainly strange. A researcher using this estimator will always conclude that the effect is identically zero regardless of the data, and for this reason, we call this estimator the *zero estimator*. The zero estimator is shown as the red vertical line in Fig 1B. Note that it is slightly off from the true value of 40 ms.

The zero-estimator is akin to a broken clock. It is correct only when the true effect is identically zero. But in the hypothetical race-priming experiment, the true effect is 40 ms, meaning that the zero estimator will fare poorly compared to consistent estimators that are calculated with large sample sizes. Sample means are known to have many favorable properties. No other unbiased estimator yields lower MSE. Moreover, sample means track the true values well and converge on them rapidly with large sample sizes.

But these statistical facts do not mean that the set of sample means outperform the zero estimator in the above environment. Fig 1C shows the distribution of root mean squared error for the difference between the sample means. Small values are desirable as they indicate less error. Note how the error is quite large across most of the distribution. With just eight observations per condition, root MSE error is about 150 ms. Let’s compare this degree of error to that for the zero estimator. The zero estimator has an error of 40 ms—if the true value is 40 ms and the estimate is zero regardless of data, then the error is always the same. This value is indicated with the red vertical line. This error is quite small. The zero estimator, while biased, is benefiting from having low variance in this environment. This phenomenon is clearly a function of effect size and sample size. As either, or both, increase, so too will the accuracy of the sample mean difference. For example, we could consider a much smaller value of *σ* for the two conditions, *σ* = 50 ms. The effect size is now much larger and we would expect the sample mean difference to be more accurate. Indeed, for this value of *σ*, we obtain a root MSE of 25 ms for the sample mean difference, with the zero estimator’s root MSE unchanged at 40 ms. Likewise, as effect size decreases, i.e., *σ* increases, we would see the opposite. For *σ* = 500 ms, the sample mean difference has root mean squared error equal to 250 ms, which is far less accurate than the zero estimator, again, unchanged at 40 ms for this example.

In this motivating example, the difference between sample means is far less accurate a measurement than the zero estimator even though the true value is nonzero. Considering these sample means, forming conclusions from them, or even reporting them overstates their measurement fidelity. They are highly inaccurate in this environment, which reflects the poor resolution from using small samples to measure small effects.

Sometimes people find it attractive to think of accuracy in terms of statistical power. Estimation accuracy is distinct from power. We need not discuss binary decisions, long term error rates, beliefs, or any other inferential system. In fact, it is a question of whether the measurement itself has the resolution to be useful, much as measurements from yardsticks are highly inaccurate for measuring the width of a hair. The reason is the same, there needs to be a match between the environment (sample and effect sizes) and the instrument (estimator).

In this example, the environment is defined by the true effect, the true variability in the data, and the sample size. In practice, two of the three elements are unknown, at least to arbitrary precision. In fact, the goal is to measure these true values, and knowing them would obviate the goal. Fortunately, we do not need to define these true values to assess accuracy. Instead, one could specify a range or the order-of-magnitude of possible effect sizes. We ask that if effects ranged on a specified scale—say 10s or 100s or milliseconds—then, what are the sample sizes needed to ensure that sample means are more accurate than the zero estimator.

In a later section we introduce a new, crude estimator that is in some cases better than the zero estimator but still not appropriate for general use. This estimator measures some important features of the data, such as the scale of possible effects, but randomizes the relations between groups or conditions. We consider sample means minimally accurate in environments where they are expected to be more accurate than this new random estimator.

Before we introduce this new random estimator, it is helpful to review some advances in modern statistical theory. These advances, which broadly go under the name of regularization in estimation, serve two purposes here. First, they help us develop our random estimator benchmark. Second, they become the basis for our tangible recommendations in tandem with the zero estimator. We recommend that modern estimators based on regularization (e.g., hierarchical Bayes, lasso, ridge, methods) be adopted broadly within the field of psychology, especially in impoverished environments (small effects and/or small sample sizes).

## Stein’s paradox and shrinkage estimation

Although it is common to think of the sample mean as a natural or obvious choice for estimation, the seminal work of Stein (1956) showed that the vector of sample means is not an admissible estimator. In other words, there exist biased estimators that always incur less MSE than the vector of sample means (for the case *p* > 2). The result was controversial at first, but accepted today and foundational in analysis under the moniker of *regularization* (e.g., lasso regression, ridge regression, Bayesian shrinkage, see [11]). Here, we review Stein’s result and its consequences for understanding the measurement properties of the sample mean.

Consider a researcher who wishes to know whether reading speed of words is affected by the color in which the words are displayed. The researcher chooses colors of red, green, yellow, and blue, and presents words, one-at-a-time, to participants. Participants are tested between-subject and see words in only one of the font colors. Note that each sample mean is an independent measure. The sample mean for red words is not informed by the responses to blue words. A seemingly natural statistical approach, then, is to calculate the sample mean for each color and the differences among them. Stein’s paradox reveals that this approach is not ideal. If one knew from the other three colors that response times tend to be on the scale of half a second or so, then this information should be used in measuring the response to red words. That is, it should be used to calibrate the estimates themselves.

**Fig 2:**
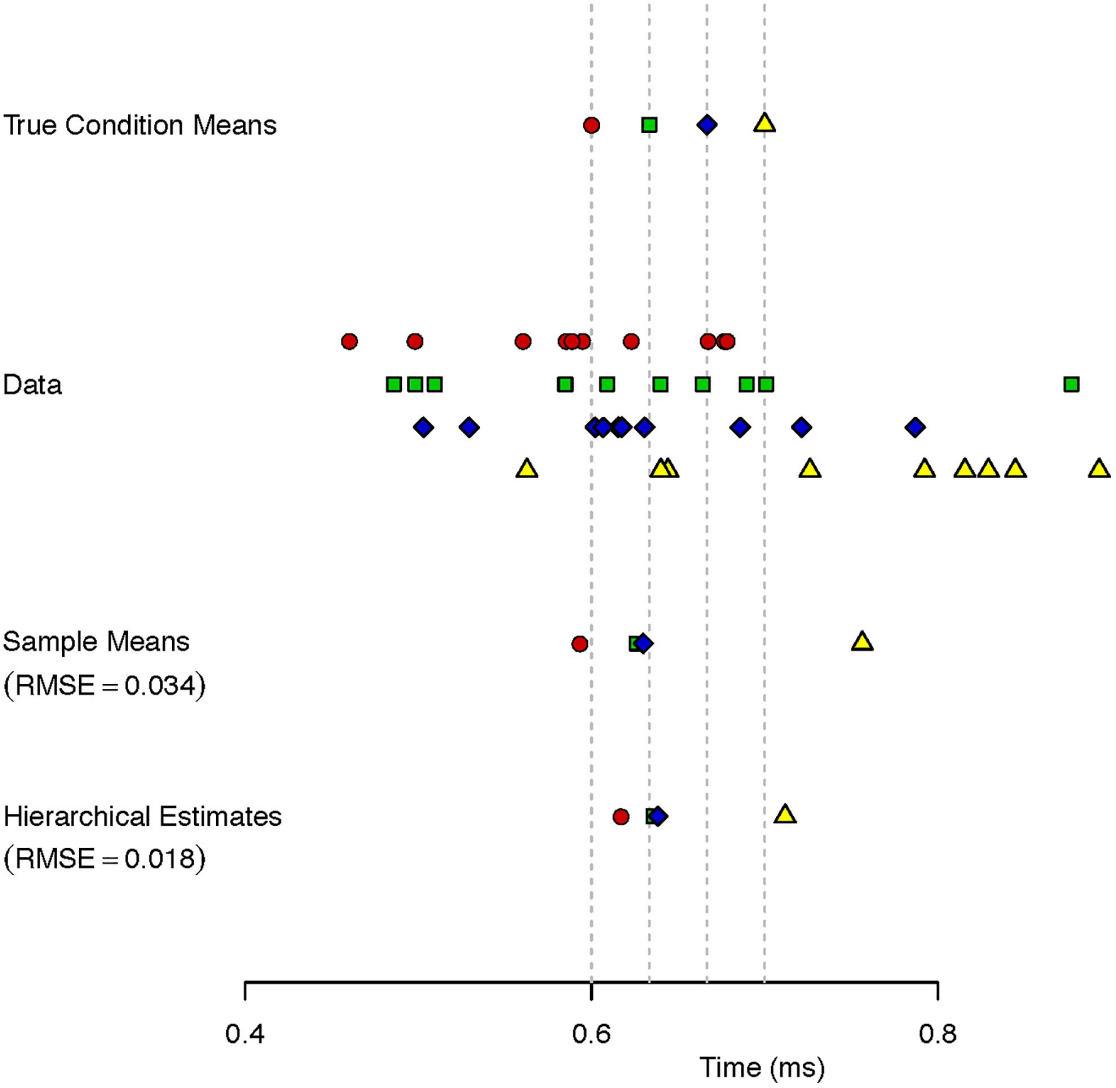
The benefits of shrinkage. True values, data, sample means, and a Bayesian hierarchical shrinkage estimates are shown. The shrinkage estimates are more accurate in this case as well as on average across repeated samples from the true values.

Fig 2 shows an example. The top row shows true condition response time means as a function of color. Red words are read more quickly than green words and so on. The data are shown next, and they are noisy draws from the respective true values. Below them are sample means, which are less perturbed from the true means than the data themselves. The bottom row shows Bayesian hierarchical-model estimates [12] which are different than the sample means. This Bayesian hierarchical model is specified as follows:

$$\begin{array}{ccc} y_{ki} & ∼ & \text{Normal}(\mu^{*}+\alpha_{k},\sigma^{2}),\\ \alpha_{k} & ∼ & \text{Normal}(0,\frac{1}{8}),\\ \pi (\mu ,\sigma^{2}) & \propto & 1/\sigma^{2}, \end{array}$$

where *y*_*k*,*i*_ is the random variable corresponding to observable responses from the *k*^*th*^ sample in the *i*^*th*^ group, μ* is the grand mean, and $\pi \left(\mu ,\sigma^{2}\right)\propto \frac{1}{\sigma^{2}}$ jointly describes a non-informative prior for μ* with equal weight on all values and a non-informative prior for variance with a flat prior on log *σ*^2^ [13]. This hierarchical model uses the combined scale of observations to “shrink” estimates toward the grand mean of all observations. That is, the hierarchical estimates will be less disperse than the sample means. This process will, in general, lower the total MSE across the four conditions by “borrowing strength” via pooling. This does not mean that individual mean estimates are necessarily more accurate, e.g., response time estimates for red words are not necessarily improved by using information about green word response times. When pooling in this manner, some individual estimates will be made more accurate with others becoming less so. On balance, the total error will be reduced.

This shrinkage property has motivated much of the work in modern estimation. Whereas pooling information always improves accuracy, the amount to pool is necessarily a function of prior assumptions. Different methods, say empirical Bayes, hierarchical Bayes, lasso, and ridge regression, vary in these prior assumptions. Even so, all are based on the above insight.

A surprising consequence of this work, known as Stein’s paradox, is that any information about the scale, no matter how small, helps improve accuracy. This argument for scaling measurements may be taken to an extreme, and doing so helps build intuition. Consider the following famous example where the goal is to measure the true mean of three populations: the weight of all hogs in Montana, the per-capita tea consumption in Taiwan, and the height of all redwood trees in California. These values could be estimated separately by getting a sample of hogs, a sample of Taiwanese households, and a sample of redwood trees and using each sample mean as an estimate of the corresponding population mean. Stein’s paradox shows that the total error of these three estimates is expected to be reduced if, rather than using sample means to estimate each individual population mean, scale information from all three samples is pooled. To be clear, pooling only helps in lowering total MSE—hog weight information does not necessarily reduce the error of the redwood tree heights estimate.

## Random estimators

We are now in a position to use shrinkage estimation to assess the accuracy of sample means. To do so, we ask if we can construct an absurd estimator, similar in spirit to the zero estimator, that outperforms sample means in common environments. If so, we consider sample means unacceptably accurate in those environments.

Here, we use randomness rather than zero. We will randomly assign measurement values to each condition so that which group means are larger and smaller, and by how much, is initially completely random. This notion of using a random benchmark to calibrate the performance of a method is standard in classification and machine learning, and is even precedented in psychology [14–16]. The innovation here is to use random estimators that have modern shrinkage properties. Although the relations between condition means are scrambled, the data are used to optimally determine the scale (i.e., calibration) of the values. The random estimator presented in the next section, though related to the class considered in [15], is both novel and ideal for assessing the accuracy of sample means.

Here is an example of how smart random estimators work: The shrinkage estimates in Fig 2 for the colors red, green, blue, and yellow are the values of 617 ms, 636 ms, 638 ms, and 712 ms. One way we can construct a random estimator with these calibrations is to randomly assign each value to a color. For example, we may assign the colors red, green, blue and yellow the values 638 ms, 636 ms, 712 ms, 617 ms. The key property of this new random estimator is that it does exactly the opposite of what psychologists value. It scrambles the exact information of interest, the relations among the conditions. For example, one is unable with this tool to truly assess whether red text is read more quickly than green text. It preserves the characteristic of no intrinsic interest, the scale of measurement, say that differences are about 30 ms. Such information, while useful in fine tuning estimates, is of no real relevance in practice to most psychologists.

We consider the sample means to be minimally accurate if they are more accurate than random estimators that scramble the relations among conditions. To characterize this, we develop a smart random estimator in the next section. This estimator leverages modern advances in shrinkage and uses the scale afforded in the data to calibrate the random relations. The argument is that if this smart random estimator, which randomizes critical information, can outperform sample means, then sample means are unacceptably accurate under those sample and effect sizes. The critical question is whether there are such environments. The answer is yes, and the environments are, unfortunately, not so rare in psychological science.

## A random estimator for condition means

The key component in a random estimator is that the relations among the condition means is random, and so, we start with random numbers. A bit of notation is helpful, and we let *p* denote the number of conditions. Let *a*_1_, *a*_2_, …, *a*_*p*_ be a sequence of independent draws from a continuous uniform distribution over the interval [−1, 1]. The choice of [−1, 1] as support for the uniform distribution was arbitrary. Due to the scaling parameter, *b*, any continuous uniform distribution whose support is bounded, convex and symmetric about 0 will yield exactly the same random estimator. We assign *a*_1_ to the first condition, *a*_2_ to the second condition, and so on. Because each of *a*_*i*_’s are random, the relations among the conditions are random and, in particular, are completely independent of any structure in the data.

Next, we use data to scale the *a*_*i*_’s. As described in the previous section, this scaling term will leverage shrinkage properties to put these random estimates on a scale appropriate to the data. We denote this scaling coefficient as *b*, and it is a single number. The random estimator for the *i*^*th*^ condition mean, denoted ${\hat{\mu }}_{i}^{re}$, is

$$\begin{array}{c} {\hat{\mu }}_{i}^{re}=\bar{G}+b\times a_{i}, \end{array}$$ (1)

where $\bar{G}$ is the observed grand mean across conditions. Here, we see the random estimator is a simple scaling of random numbers. The value of *b*, discussed subsequently, can be positive or negative, depending on the data. In either case, the order among condition estimates is random, that is, it reflects the random relations among the random numbers *a*_1_, …, *a*_*p*_.

Remaining is the construction of *b*. We use a least-squares argument with optimized shrinkage compared to sample means—see S1 File for a full derivation. The scalar *b* is constructed to insure that ${\hat{\mu }}^{re}$ minimizes squared error subject to the above random assignment constraints. The resultant is

$$\begin{array}{c} b=(\frac{p-\sqrt{p(p-1)}}{\sqrt{\sum_{i=1}^{p}a_{i}^{2}}}\sum_{i=1}^{p}a_{i}\alpha_{i}), \end{array}$$

where $\alpha_{i}={\bar{y}}_{i}-\bar{G}$, with ${\bar{y}}_{i}$ defined to be the sample mean for the *i*^*th*^ condition. Although the scaling term *b* makes full use of the data, which is ‘smart’ from an estimation perspective, this scaling parameter is quite limited. It can either: (1) leave the random ordering of the *a*_*i*_’s unchanged (if *b* > 0) or (2) it can completely reverse the random ordering (if *b* < 0). Hence, the order of estimates is randomized, although *not* uniformly as the *b* term can reverse the random ordering according to the data. Because of this, the *p* = 2 case does not randomize order at all compared to sample means. For this case, the zero estimator comparison may be more appropriate, although, as we later discuss, the zero and random estimators yield similar sample size recommendations. As *p* increases, the correlation between the order of the estimates generated by the random estimator and the order generated by sample means quickly goes to zero.

Whether the random estimator incurs less MSE than sample means depends upon the environment, i.e., sample size and effect size. As a simple example of how to calculate our random estimator, consider the hypothetical data in the previous example on naming words as a function of display color. The sample means for this set of data are equal to: ${\bar{y}}_{1}=593$ ms, ${\bar{y}}_{2}=626$ ms, ${\bar{y}}_{3}=630$ ms, and ${\bar{y}}_{4}=756$ ms, for Conditions 1-4 respectively. We refer to this pattern of means as the observed pattern of treatment effects. Now, let’s consider what the random estimator would yield as estimates of the population means for these data. First, we draw four uniform random numbers from the [−1, 1] interval that correspond to the four conditions. For Condition 1, we obtain -.190, for Condition 2, we obtain -.973, for Condition 3, we obtain.823, and for Condition 4 we obtain.600. Note that once a random number has been drawn for a condition, we cannot shuffle or re-assign it to another condition after the fact. We calculate the scale factor of *b* as 21.5, which portends quite a bit of shrinkage. Using this value, the random estimates are: $\mu_{1}^{re}=647$ ms, $\mu_{2}^{re}=630$ ms, $\mu_{3}^{re}=669$ ms, $\mu_{4}^{re}=664$ ms. Note that all four estimates are close to the grand mean. Recall that the order of the sample means was: Condition 1 < Condition 2 < Condition 3 < Condition 4. In contrast, the random estimator now assigns the order: Condition 2 < Condition 1 < Condition 4 < Condition 3. This is the same random order that was produced by the random *a*_*i*_ draws. Note also that the relative distance between the random estimates has also been randomized; the estimates for Conditions 3 and 4 are quite close, in contrast to the sample means, where ${\bar{y}}_{4}$ is relatively farther from ${\bar{y}}_{3}$.

This illustration was based on a single sample. Of course, the stronger argument is what may be expected across all samples for an environment. Perhaps the most straightforward approach to generalization is to simply simulate many samples from the same environment. As a simulation example, we will define a particular environment and compare the accuracy of sample means to the random estimator directly. As before, consider four treatment conditions under a balanced design. Let the four population means be equal to: *μ*_1_ = 600 ms, *μ*_2_ = 620 ms, *μ*_3_ = 640 ms, and *μ*_4_ = 660 ms. For this simulation we will assume that the dependent variable being measured is normally distribution with a standard deviation of 100 ms. This combination of means and variance gives an overall effect size of *f*^2^ = .05, which is a “small” effect according to the Cohen conventions [17]. A single repetition will consist of the following steps: (1) generate ten samples (*n* = 10) from each of the four distributions, (2) calculate the four sample means and our random estimates, and (3) calculate the squared error from each set of estimates and the four population means. By repeating steps (1)-(3) many times and averaging the squared error for each estimation method we can obtain an estimate of MSE. To arrive at a good estimate, we carried out 10,000 repetitions. The R code used to generate all of the figures is publicly available at https://figshare.com/s/0c2bbcab9e5ce4e8e7fa.

**Fig 3:**
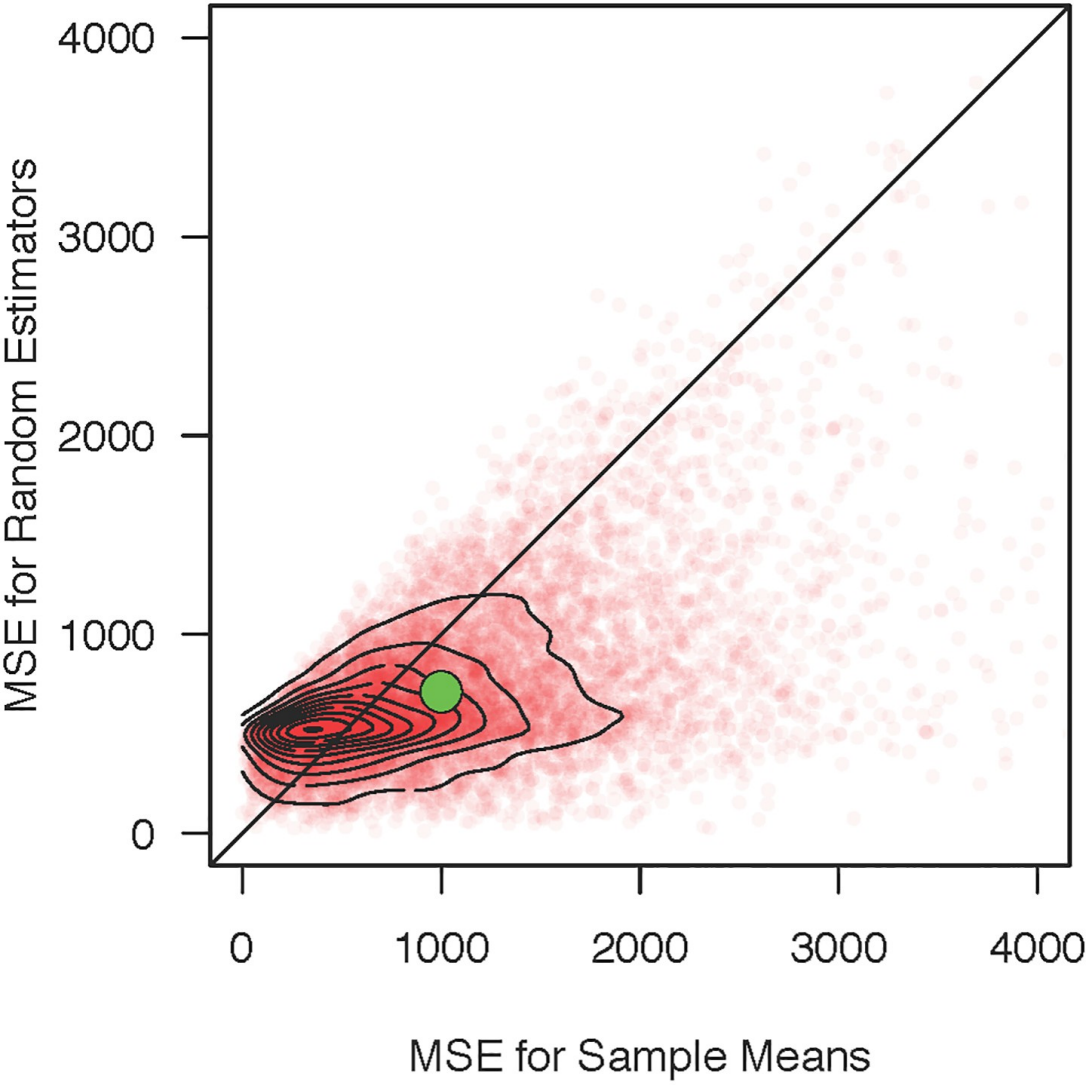
Mean-square error for the sample mean and the random estimator for 10 observations in each of four conditions. Each point shows the result from a simulation repetition, and there are 10,000 such repetitions. Contours are bivariate kernel density estimates. The large point is the mean of MSEs across repetitions. This mean for sample means is 40% larger than the mean for the random estimator, and 63% of samples show larger MSE for the sample mean.

Fig 3 shows the mean-squared error in estimation for each repetition, and the big green dot shows the mean over all repetitions. The mean error is 30% larger for the sample mean than for the random estimator, and, moreover, 62% of the samples show larger error for the sample mean than for the random estimator. Given these results, it is hard to take the sample means seriously when they are more error prone than the random estimator. In this sense, sample means are insufficiently accurate.

## Planning and evaluating designs

The main advantage of zero- and random-estimators is that they provide a benchmark for using sample means to evaluate the ordering of conditions. If these nonsense estimators with no or randomized orders are expected to do a better job of accounting for the structure in data than sample means, then we argue that sample means are not sufficiently accurate. For simple designs, say one-way, between-group ANOVA designs, it is possible to derive expressions for the minimum sample size per condition for which sample means are indeed more accurate than the zero- or random-estimator.

The key input to these expressions for minimum sample size is a minimum Cohen noncentrality effect-size measure one wishes to resolve, *f*^2^. This measure, along with sample size and number of conditions, defines the environment. The noncentrality measure is

$$\begin{array}{c} f^{2}=\frac{\frac{1}{p}\sum_{i=1}^{p}{\left(\mu_{i}-\mu^{*}\right)}^{2}}{\sigma^{2}}, \end{array}$$

where μ* is the true grand mean given by $\mu^{*}=\left(\sum_{i}^{p}\mu_{i}\right)/p$ and *p* is the number of conditions. For calibration purposes, Cohen recommends using *f*^2^ values of .01, .0625, and .16 as emblematic of small, medium, and large effects, respectively. These values for *f*^2^ are analogs to the values of .2, .5, and .8, respectively, for the better known *d* measure. The *f*^2^ measure is also related to *R*^2^, the proportion of variance accounted for by the condition structure as

$$\begin{array}{c} f^{2}=\frac{R^{2}}{1-R^{2}}. \end{array}$$

Expressing a minimum sample size for sample mean accuracy works as follows: First the researcher chooses a value of *f*^2^ to be resolved in an experiment. Then, the minimum sample size per condition needed to insure that on average sample means are more accurate than the random estimator can be obtained via the following result.

Proposition 1. *Let MSE_re_ denote the MSE of the random estimator*, ${\hat{\mu }}_{i}^{re},i\in \left\{1,2,\ldots ,p\right\}$. *Let MSE_sm_ denote the MSE of the vector of sample means*, ${\bar{y}}_{i},i\in \left\{1,2,\ldots ,p\right\},$. *The ratio* $\frac{MSE_{re}}{MSE_{sm}}$ *is less than 1, if, and only if*,

$$\begin{array}{c} n<\frac{\sqrt{p(p-1)}}{pf^{2}}, \end{array}$$ (2)

*where n is the sample size per condition in the experimental design*.

A proof is provided in S1 File. As an example, if there are 5 conditions and the minimum *f*^2^ to be resolved is.05, then the value of *n* needed, at a minimum, for sample means to be more accurate than the random estimator is 17.9, which rounds up to 18. Similarly, the minimum sample size so that the sample mean is more accurate than the zero estimator is as follows. Recall that the zero estimator simply uses the grand mean, $\bar{G}$, as the estimate for each population mean.

Proposition 2. *Let MSE_z_ denote the MSE of the zero estimator*, $\bar{G}$. *Let MSE_sm_ denote the MSE of the vector of sample means*, ${\bar{y}}_{i},i\in \left\{1,2,\ldots ,p\right\},$. *The ratio* $\frac{MSE_{z}}{MSE_{sm}}$ *is less than 1, if, and only if*,

$$\begin{array}{c} n<\frac{\left(p-1\right)}{pf^{2}}. \end{array}$$ (3)

**Fig 4:**
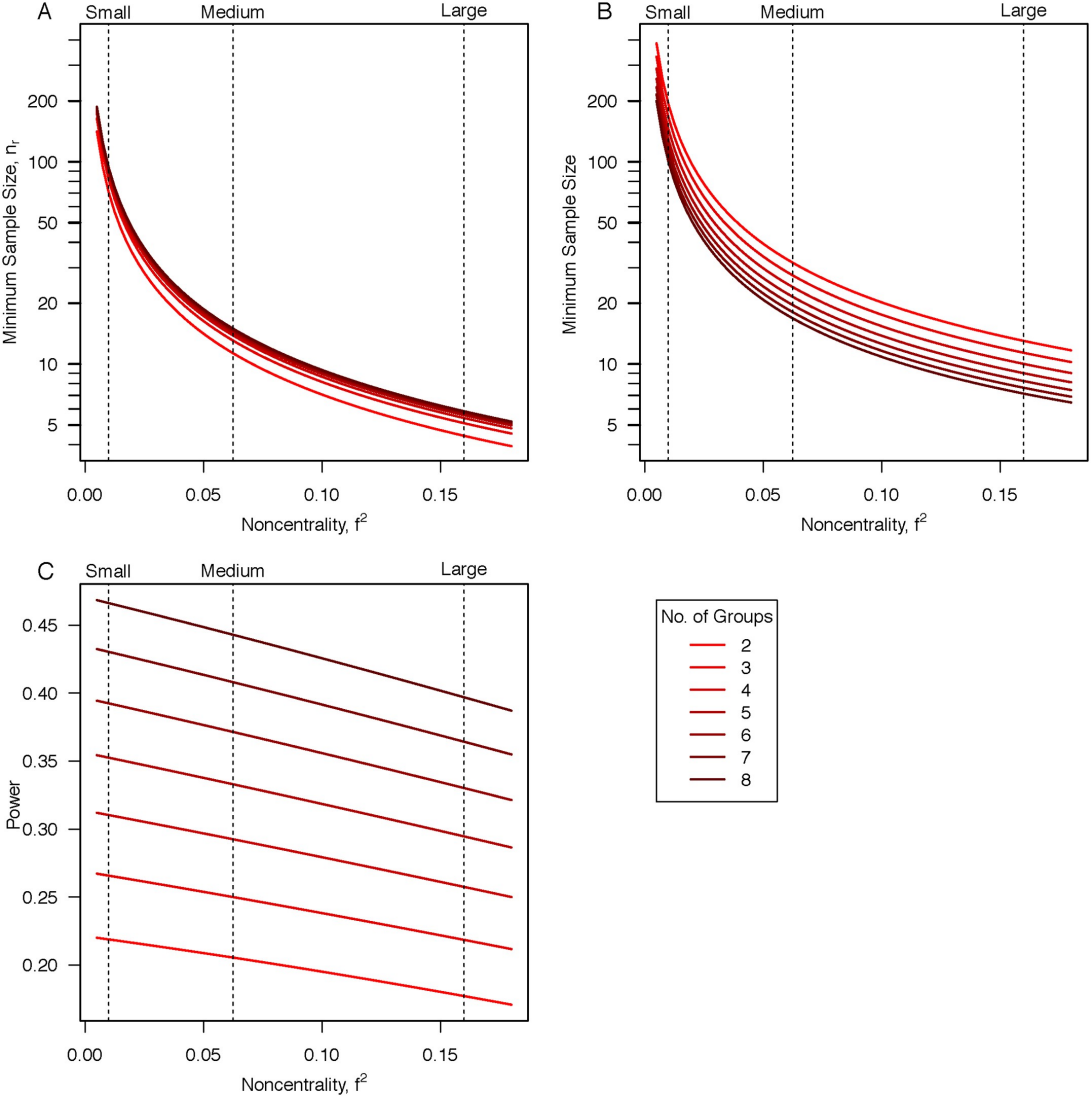
**A**. Minimum sample size (per condition) for minimal acceptable accuracy of the sample mean as a function of effect size *f*^2^ and the number of conditions. One of the more noteworthy aspects is that the needed sample size per condition increases with the number of conditions. **B**. Minimum sample size (per condition) for a power of 50% at the 5% level. The needed sample size per condition decreases with the number of conditions. **C**. The power at the 5% level for the minimum sample size for minimal sample mean accuracy. Surprisingly, commonly powered designs often yield unacceptably accurate sample means.

The proof is trivial. It is easy to see that the right-hand side of inequality (2) is larger than the right-hand side of inequality (3) by a factor of $\sqrt{\frac{p-1}{p}}$. For this reason, we focus on the sample size requirements for the random estimator. Let *n*_*r*_ be the minimum sample size per condition such that *MSE*_*re*_ is greater than *MSE*_*sm*_. Fig 4A shows the dependency of *n*_*r*_ on *f*^2^ for experiments with 2, 4, 6, and 8 conditions. The minimum sample sizes per condition for minimal accuracy can be quite large, say around 80 observations per condition to resolve small effects. In designs with smaller sample sizes, random estimators will outperform sample means on average.

Recall that MSE for any estimator can be decomposed into the sum of squared bias and total variance. For the random estimator, it is straightforward to see how it outperforms sample means under the conditions we specify. The squared bias of the random estimator is equal to

$$\begin{array}{c} \sum_{i=1}^{p}{\left(E\left[{\hat{\mu }}_{i}^{re}\right]-\mu_{i}\right)}^{2}=\left(p-1\right)f^{2}\sigma^{2},\\ =\frac{p-1}{p}\sum_{i=1}^{p}{\left(\mu_{i}-\mu^{*}\right)}^{2}. \end{array}$$

As the squared distance between the population means becomes smaller, all else equal, so does the squared bias of the random estimator. The random estimator incurs this bias penalty but outperforms sample means, under the conditions we specify, by having less total variance. The total variance of the random estimator is equal to

$$\begin{array}{c} \sum_{i=1}^{p}Var\left({\hat{\mu }}_{i}^{re}\right)=\frac{\sigma^{2}\left(p+\left(p-1\right){\left(p-\sqrt{p^{2}-p}\right)}^{2}\right)}{pn}+\left(p-1\right)\left(2p-1-2\sqrt{p^{2}-p}\right)f^{2}\sigma^{2}. \end{array}$$

While not immediately obvious, this term is much smaller than the total variance of sample means, which is equal to $\frac{p\sigma^{2}}{n}$, for modest effect sizes, *f*^2^. In this way, the random estimator trades an increase in squared bias for a reduction in total variance—which, under the conditions we specified, allow it to incur less MSE than sample means.

For comparison, Fig 4B shows the minimum sample size per condition to maintain 50% power (at the 5% Type I level) in a one-way ANOVA *F*-test. As can be seen, these minimum sizes are larger than that needed for minimal accuracy, indicating that minimal accuracy is a low bar. Also evident is that the number of conditions affects minimum sample size differently for minimal accuracy than for power. The minimum sample size increases with condition size for minimal accuracy but decreases for power (indeed, increasing the number of conditions leads to a more stable estimate of within-condition variance). As a result, with several conditions, it may be possible to have high powered designs that have insufficiently accurate sample means. In such cases, it is hard to see how the ensuing inferences are sound even with high power. Overall, increasing the number of conditions (while maintaining sample size per condition) makes it easier to detect differences, but it makes it harder measure the ordering relationships accurately.

The above minimum sample size expressions are general in that they hold no matter how the dependent variable is distributed. The sole assumption is the familiar one of homogeneity where variance is assumed constant across conditions. In the online supplement we provide simulation code that examines our sample size recommendations when the homogeneity of variance assumption is violated for *p* = 3, 5. We find our primary result, Proposition 1, to be very robust to homogeneity of variance violations.

### How often are sample means insufficiently accurate?

One might ask: how often do psychologists utilize sample means that are insufficiently accurate? One way to answer this question is to examine statistical power values for sample sizes that are at the threshold for minimal sample mean accuracy. Fig 4C shows the statistical power (at the 5% Type-I level) for a one-way ANOVA for *n*_*r*_, the minimum sample size per condition for sample means to outperform our random estimator. It is clear from this graph that sample means are unacceptably accurate for non-trivial power values, especially when there are many conditions.

How well powered are studies in the literature? Although this is a difficult question, there are several model-based meta-analyses that estimate power across different areas of psychology. Average statistical power in many areas of psychology is at or below.5, with average power from studies with small effects hovering around.17 [3, 18–20]. A recent study by Szucs and Ioannidis [21] examined more than 100,000 statistical records from about 10,000 cognitive neuroscience and psychology papers published within the past five years. They found that the median power to detect small effect sizes was .12—which clearly equates to an inaccurate sample mean environment—see Fig 4C. We argue that sample means should not be used in such environments. Instead, it is imperative for researchers to use more sophisticated methods that incorporate modern inference and regularization when investigating such effects.

## General discussion

Sample means are measurement instruments, and like physical measurements, are appropriate in specific environments. In low-resolution environments, sample means are outperformed by smart random estimators that scramble the relations among conditions. In such situations, it seems that using sample means to characterize treatment effects is inappropriate, since estimates that obscure treatment effects more accurately characterize the data. Unfortunately, low-resolution describes the typical combination of sample and effect sizes in many areas of psychological research.

### Why mean-squared error as metric of accuracy?

Throughout this article, we have used MSE (or root MSE) as our measure of estimation accuracy. This choice is for four important reasons: First, the usage of MSE as an accuracy metric is ubiquitous within the field of statistics and is treated often as a gold standard. Indeed, it is well-known that sample means are the best linear unbiased estimator by exactly this criterion. Second, nearly all of the major statistical tests used in psychology are based on a squared error metric, e.g., ANOVA. Third, using MSE makes our results distribution-free. This is not a minor point. Our main results (Propositions 1 and 2) hold whether the dependent variable is normally distributed or not. Fourth, MSE allows for an interpretable decomposition of accuracy. Since sample means are unbiased, MSE is simply the variance of the sample mean estimates. Said in plain language, sample means are so variable, under the conditions we identify, that biased, nonsensical estimators outperform them by the simple virtue of being less variable.

### Sample means are not ideal

Modern estimation theory stresses shrinkage estimators over sample means, and examples include Bayesian hierarchical estimators, lasso estimators, and ridge estimators. These estimators make use of condition relational information, like the sample mean, and scale information, like the random estimator. Because they use information wisely, these modern estimators outperform sample means and random estimators in all measurement environments. They are better measurement tools. These regularized measures are increasingly common in sophisticated data analysis [22] precisely because they are more accurate in these environments.

One of our main conclusions is that researchers should avoid reporting sample means in favor of more sophisticated estimates. Two issues remain: First, and perhaps of lesser importance, is that modern estimation requires some calibration for shrinkage. Almost all regularization techniques have a smoothing, variability, or banding parameter. This parameter can be set based on the analysts judgment or from default procedures. One example of such a default procedure is the usage of empirical Bayes where empirical estimates across units calibrate the shrinkage. Another example is the use of unit-information priors such as that underlying the Bayesian interpretation of BIC [23] This is a highly active area of research and new Bayesian estimators can employ a wide range of regularization techniques [24] The second issue is more consequential. Researchers are comfortable with sample means. And why shouldn’t they be? We have all been taught the arithmetic average since grade school. The sample mean is the most privileged measure of central tendency, and it takes center stage over its less glorious siblings, median and mode. Yet, the performance of the random estimators in certain environments, and the superiority of modern estimators more generally, serve to undermine this privileged and natural status. In our view, sample means, sample medians, and modes should not be taught as descriptives. Instead, they should be taught within the context of estimation where statistics serve not to describe but to estimate unknown parameters. Different estimators have different desiderata pertaining to bias, efficiency and loss. Although this approach is more complicated and difficult than putting the sample mean on a pedestal, it seems necessary for clear scientific thought.

## Supporting information

S1 File Mathematical appendix. (PDF)
