# Correction: Estimation accuracy in the psychological sciences

**DOI:** 10.1371/journal.pone.0213461

**Published:** 2019-02-28

**Authors:** Clintin P. Davis-Stober, Jason Dana, Jeffrey N. Rouder

There are errors in S1 File. Due to a LaTex compile error, some equation numbers incorrectly appear as question marks. Please view the correct S1 File below.

## Supporting information

S1 File Mathematical appendix. (PDF)
