# Supplementary material for: Estimation accuracy in the psychological sciences
Source: PLoS One. 2018 Nov 26;13(11):e0207239. doi: 10.1371/journal.pone.0207239 (PMC6261010; doi:10.1371/journal.pone.0207239)
Supplement: S1 File — (PDF) [file pone.0207239.s001.pdf]

Estimation accuracy in the psychological sciences

Clintin P. Davis-Stober[1,*], Jason Dana[2], Jeffrey N. Rouder[3]

1 Department of Psychological Sciences, University of Missouri, Columbia, MO, USA
2 Yale School of Management, Yale University, New Haven, CT, USA
3 Department of Cognitive Sciences, University of California, Irvine, Irvine, CA, USA

* stoberc@missouri.edu

Supporting information

S1 Appendix. This Supporting Information file is organized as follows. First, we define the random estimator under consideration. This is followed by a primary result of the paper, in which we precisely define the conditions under which this random estimator incurs less mean squared error than sample means. The remainder of the Supporting Information is devoted to a full proof, complete with auxiliary definitions and derivations.

Definition 1. Let $y_i, i \in \{1, 2, \ldots, p\}$, be a set of p-many independent random variables with corresponding population means, $\mu_i, i \in \{1, 2, \ldots, p\}$, and variance σ^2. Assume that $p \geq 2$. Assume n many independent and identical draws from each $y_i, i \in \{1, 2, \ldots, p\}$. Let $a_i, i \in \{1, 2, \ldots, p\}$, be a set of weights obtained from independent (continuous) uniform random draws over the closed interval $[-1, 1]$. Following standard "effect" notation [?], we will work with the difference between each sample mean and the overall grand mean, i.e., let $\alpha_i = \overline{y}_i - \overline{G}$ where $\overline{y}_i$ is the usual sample mean for the ith group, and $\overline{G}$ is the observed grand mean, defined and calculated in the usual way from the experimental data. Our *random estimator* is defined as follows:

$$\hat{\mu}_i^{re} = a_i \left(\frac{p - \sqrt{p(p-1)}}{\sqrt{\sum_{i=1}^{p} a_i^2}} \sum_{i=1}^{p} a_i \alpha_i \right) + \overline{G}, \qquad i \in \{1, 2, \ldots, p\}.$$

Proof of Proposition 1. As an intermediary step to proving the Main Result, we will first consider an estimator that is the least-squares solution for the scaling factor, b (see (**??**)), subject to the random constraints implied by the weights $a_i, i \in \{1, 2, \ldots, p\}$, and solve for its mean squared error. This *random least squares estimator* is defined as follows:

$$\hat{\mu}_i^{rnd} = a_i \left(\frac{1}{\|\boldsymbol{a}\|} \sum_{i=1}^{p} a_i \alpha_i \right) + \overline{G}, \qquad i \in \{1, 2, \ldots, p\}. \tag{1}$$

Mean squared error is defined as $E[\sum_{i=1}^{p} (\hat{\mu}_i - \mu_i)^2]$, where $\hat{\mu}_i$ is an estimator of μ_i. We prove the result by deriving $E[\sum_{i=1}^{p} (\hat{\mu}_i^{rnd} - \mu_i)^2]$ and solving the inequality $E[\sum_{i=1}^{p} (\hat{\mu}_i^{rnd} - \mu_i)^2] < E[\sum_{i=1}^{p} (\overline{y}_i - \mu_i)^2]$. To simplify notation, and without loss of generality, we assume that $\|\boldsymbol{a}\| = 1$.

Recall that for any estimator, $E[\sum_{i=1}^{p} (\hat{\mu}_i - \mu_i)^2] = \sum_{i=1}^{p} Var(\hat{\mu}_i) + \sum_{i=1}^{p} (E[\hat{\mu}_i] - \mu_i)^2$, where $Var(\hat{\mu}_i)$ denotes the variance of $\hat{\mu}_i$. Solving for $\sum_{i=1}^{p} Var(\hat{\mu}_i^{rnd})$, we obtain,

$$\sum_{i=1}^{p} Var(\hat{\mu}_i^{rnd}) = \sum_{i=1}^{p} \left(E[(\hat{\mu}_i^{rnd})^2] - (E[\hat{\mu}_i^{rnd}])^2 \right),$$

$$= \sum_{i=1}^{p} \left(E[a_i^2(\sum_{i=1}^{p} a_i(\overline{y}_i - \frac{1}{p}\sum_{i=1}^{p}\overline{y}))^2 + 2a_i(\sum_{i=1}^{p} a_i(\overline{y}_i - \frac{1}{p}\sum_{i=1}^{p}\overline{y}))(\frac{1}{p}\sum_{i=1}^{p}\overline{y}_i) + \frac{1}{p^2}(\sum_{i=1}^{p}\overline{y})^2] - (E[\hat{\mu}_i^{rnd}])^2 \right),$$

$$= \sum_{i=1}^{p} \left(E[a_i^2(\sum_{i=1}^{p} a_i(\overline{y}_i - \frac{1}{p}\sum_{i=1}^{p}\overline{y}))^2] + E[2a_i(\sum_{i=1}^{p} a_i(\overline{y}_i - \frac{1}{p}\sum_{i=1}^{p}\overline{y}))(\frac{1}{p}\sum_{i=1}^{p}\overline{y}_i)] + E[\frac{1}{p^2}(\sum_{i=1}^{p}\overline{y}_i)^2] - \right.$$
$$\left. \frac{\mu_i^2}{p^2} - \frac{2(p-1)}{p^3}\mu_i(\sum_{i=1}^{p}\mu_i) - \frac{(p-1)^2}{p^4}(\sum_{i=1}^{p}\mu_i)^2 \right),$$

$$= \sum_{i=1}^{p} \left(E[a_i^2(\sum_{i=1}^{p} a_i(\overline{y}_i - \frac{1}{p}\sum_{i=1}^{p}\overline{y}))^2] + E[2a_i(\sum_{i=1}^{p} a_i(\overline{y}_i - \frac{1}{p}\sum_{i=1}^{p}\overline{y}))(\frac{1}{p}\sum_{i=1}^{p}\overline{y}_i)] + \frac{\sigma^2}{pn} + (\frac{1}{p}\sum_{i=1}^{p}\mu_i)^2 - \right.$$
$$\left. \frac{\mu_i^2}{p^2} - \frac{2(p-1)}{p^3}\mu_i(\sum_{i=1}^{p}\mu_i) - \frac{(p-1)^2}{p^4}(\sum_{i=1}^{p}\mu_i)^2 \right).$$

Since $\sum_{i=1}^{p} E[2a_i(\sum_{i=1}^{p} a_i(\overline{y}_i - \frac{1}{p}\sum_{i=1}^{p}\overline{y}))(\frac{1}{p}\sum_{i=1}^{p}\overline{y}_i)] = \frac{\sigma^2}{pn} + (\frac{1}{p}\sum_{i=1}^{p}\mu_i)^2 - \frac{\sigma^2}{pn} - (\frac{1}{p}\sum_{i=1}^{p}\mu_i)^2 = 0$, we can further simplify and carry out summation,

$$\sum_{i=1}^{p} Var(\hat{\mu}_i^{rnd}) = \left(E[\sum_{i=1}^{p} a_i^2(\sum_{i=1}^{p} a_i(\overline{y}_i - \frac{1}{p}\sum_{i=1}^{p}\overline{y}))^2] \right) + \frac{\sigma^2}{n} + \frac{1}{p^3}(\sum_{i=1}^{p}\mu_i)^2 - \frac{1}{p^2}\sum_{i=1}^{p}\mu_i^2.$$

Since $\sum_{i=1}^{p} a_i^2 = 1$,

$$\sum_{i=1}^{p} Var(\hat{\mu}_i^{rnd}) = \left(E[(\sum_{i=1}^{p} a_i(\overline{y}_i - \frac{1}{p}\sum_{i=1}^{p}\overline{y}))^2] \right) + \frac{\sigma^2}{n} + \frac{1}{p^3}(\sum_{i=1}^{p}\mu_i)^2 - \frac{1}{p^2}\sum_{i=1}^{p}\mu_i^2.$$

Since a_i is uncorrelated with $a_j, (i \neq j)$, and all $a_i, i \in \{1, 2, \ldots, p\}$ values are pairwise independent of $\overline{y}_i, i \in \{1, 2, \ldots, p\}$, we obtain,

$$\sum_{i=1}^{p} Var(\hat{\mu}_i^{rnd}) = \frac{1}{p} \left(\sum_{i=1}^{p} E[(\overline{y}_i - \frac{1}{p}\sum_{i=1}^{p}\overline{y}_i)^2] \right) + \frac{\sigma^2}{n} + \frac{1}{p^3}(\sum_{i=1}^{p}\mu_i)^2 - \frac{1}{p^2}\sum_{i=1}^{p}\mu_i^2,$$

$$= \frac{1}{p} \left(\frac{p\sigma^2}{n} + (\sum_{i=1}^{p}\mu_i^2) - \frac{\sigma^2}{n} - \frac{1}{p}(\sum_{i=1}^{p}\mu_i)^2 \right) + \frac{\sigma^2}{n} + \frac{1}{p^3}(\sum_{i=1}^{p}\mu_i)^2 - \frac{1}{p^2}\sum_{i=1}^{p}\mu_i^2,$$

$$= \frac{(2p-1)\sigma^2}{pn} + \frac{(p-1)}{p^2} \left((\sum_{i=1}^{p}\mu_i^2) - \frac{1}{p}(\sum_{i=1}^{p}\mu_i)^2 \right).$$

Next, we solve for the sum of squared bias term, $\sum_{i=1}^{p}(E[\hat{\mu}_i^{rnd}] - \mu_i)^2$. We obtain,

$$\sum_{i=1}^{p}(E[\hat{\mu}_i^{rnd}] - \mu_i)^2 = \sum_{i=1}^{p} \left(\frac{\mu_i}{p} + \frac{(p-1)}{p^2}(\sum_{i=1}^{p}\mu_i) - \mu_i \right)^2,$$

$$= \frac{(p-1)^2}{p^2}(\sum_{i=1}^{p}\mu_i^2) - \frac{(p-1)^2}{p^3}(\sum_{i=1}^{p}\mu_i)^2.$$

Combining terms gives the following mean squared error value,

$$E[\sum_{i=1}^{p}(\hat{\mu}_i^{rnd} - \mu_i)^2] = \sum_{i=1}^{p} Var(\hat{\mu}_i^{rnd}) + \sum_{i=1}^{p}(E[\hat{\mu}_i^{rnd}] - \mu_i)^2 =$$

$$\frac{(2p-1)\sigma^2}{pn} + \frac{(p-1)}{p} \left((\sum_{i=1}^{p}\mu_i^2) - \frac{1}{p}(\sum_{i=1}^{p}\mu_i)^2 \right),$$

which completes the proof of this intermediary step. $\square$.

Building upon the general idea of shrinkage estimation, we consider the random least squares estimator with the first term multiplied by a fixed scalar value, k, which will function as our shrinkage parameter (recall we set $\|\boldsymbol{a}\| = 1$),

$$\hat{\mu}_i^{rk} = a_i \left(\sum_{i=1}^{p} a_i \alpha_i \right) k + \overline{G}, \qquad i \in \{1, 2, \dots, p\}, \tag{2}$$

where $k \in [0, 1]$. To clarify, k is a variable we are introducing for the purposes of deriving our benchmark estimator defined in Definition 1 and (**??**). Clearly, when $k = 1$ the estimator defined in Equation (**??**) is the random least squares estimator, likewise, when $k = 0$ the estimator is simply the grand mean. Following the above proof for the mean squared error of the random least squares estimator, it is routine to show that the mean squared error for the estimator defined in Equation (**??**) is equal to the following:

$$MSE_{\hat{\mu}^{rk}}(k) = \frac{\sigma^2((p-1)k^2 + p)}{pn} + \frac{(p - 2k + k^2)}{p} \left((\sum_{i=1}^{p} \mu_i^2) - \frac{1}{p} (\sum_{i=1}^{p} \mu_i)^2 \right). \tag{3}$$

Similarly, it is routine to show that the estimator defined in Equation (**??**) incurs less mean squared error than the vector of sample means if, and only if,

$$n < \frac{(p-1)(p-k^2)}{p(p + (k-2)k) f^2}. \tag{4}$$

We can consider the right-hand side of Inequality (**??**) as a polynomial in k. We solve for the unique value of k, $k \in [0, 1]$, that maximizes the right-hand side of Inequality (**??**), following the usual method of taking the derivative of $z(k) := \frac{(p-1)(p-k^2)}{p(p+(k-2)k)f^2}$ and solving $z(k)' = 0$. After checking the usual optimality conditions, we obtain $k = p - \sqrt{p(p-1)}$. Substituting this value into Equation (**??**), we obtain the random estimator defined in Definition 1 and (**??**). The random estimator incurs less mean squared error than the vector of sample means if, and only if,

$$E[\sum_{i=1}^{p} (\hat{\mu}_i^{re} - \mu_i)^2] < E[\sum_{i=1}^{p} (\overline{y}_i - \mu_i)^2],$$

$$\iff \qquad \frac{\sigma^2(p + (p-1)(p - \sqrt{p^2 - p})^2)}{pn} + 2f^2\sigma^2(p-1)(p - \sqrt{p^2 - p}) < \frac{p\sigma^2}{n},$$

$$\iff \qquad 2f^2\sigma^2(p-1)(p - \sqrt{p^2 - p}) < \frac{p^2\sigma^2}{pn} - \frac{\sigma^2(p + (p-1)(p - \sqrt{p^2 - p})^2)}{pn},$$

$$\iff \qquad 2f^2\sigma^2(p-1)(p - \sqrt{p^2 - p}) < \frac{\sigma^2(p^2 - p - (p-1)(p - \sqrt{p^2 - p})^2)}{pn},$$

$$\iff \qquad 2f^2\sigma^2(p-1)(p - \sqrt{p^2 - p}) < \frac{\sigma^2(-2p^3 + 4p^2 - 2p + 2(p^2 - p)\sqrt{p^2 - p})}{pn},$$

$$\iff \qquad 2f^2\sigma^2(p-1)(p - \sqrt{p^2 - p}) < \frac{2\sigma^2(p-1)(\sqrt{p^2 - p} - (p-1))}{n},$$

$$\iff \qquad n < \frac{\sqrt{p^2 - p} - (p-1)}{(p - \sqrt{p^2 - p})f^2},$$

$$\iff \qquad n < \frac{\sqrt{p(p-1)}}{pf^2},$$

which completes the proof. $\square$.

References

1. Maxwell SE, Delaney HD. Designing experiments and analyzing data: A model comparison perspective. Routledge; 2003.
